# Effect of age on eggshell color and Haugh unit of eggs, protoporphyrin IX and ovomucin deposition and related gene expression in Hyline Brown laying hens

**DOI:** 10.5713/ab.250904

**Published:** 2026-03-11

**Authors:** Huan Xu, Yueping Chen, Xiaowei Sun, Yanmin Zhou, Chao Wen

**Affiliations:** 1 College of Animal Science and Technology, Nanjing Agricultural University, Nanjing, China

**Keywords:** Age, Eggshell Color, Haugh Unit, Laying Hen, Lipid Peroxidation

## Abstract

**Objective:**

This study was conducted to investigate the effects of age on eggshell color, Haugh unit, and underlying mechanism in Hyline Brown laying hens.

**Methods:**

A total of 192 Hyline Brown laying hens at 165 (D165 group), 307 (D307 group), or 475 (D475 group) days of age were divided into 3 groups with 8 replicates of 8 birds each, and fed the same diet for three weeks.

**Results:**

Compared to the D165 group, the D307 and D475 groups had higher (p<0.05) eggshell L* value and yolk ratio but lower (p<0.05) albumin ratio; the D475 group had higher (p<0.05) eggshell b* value but lower (p<0.05) albumin height, Haugh unit and eggshell a* value. The D165 group had higher (p<0.05) eggshell protoporphyrin IX content and ovomucin content in eggs than the D307 and D475 groups. The D165 group showed higher (p<0.05) total superoxide dismutase activity and glutathione content in eggshell gland compared to the D307 group. The malondialdehyde contents in serum, eggshell gland and magnum were higher in the D475 group than in the D165 group (p<0.05). The D307 and D475 groups had lower (p<0.05) mRNA levels of genes related to pigment synthesis and transport (coproporphyrinogen oxidase, aminolevulinic acid synthase-1 and ATP-binding cassette subfamily G member 2) than the D165 group. The D475 group had lower (p<0.05) mRNA levels of genes related to albumin synthesis (α-ovomucin and lysozyme) than the D165 and D307 groups.

**Conclusion:**

As hens aged, the deterioration of eggshell color and Haugh unit of eggs resulted from decreased gene expression and secretion of protoporphyrin IX and ovomucin, which could be attributed to increased lipid peroxidation in eggshell gland and magnum.

## INTRODUCTION

It has been discovered that with the extension of the laying cycle, the decline of egg quality such as decreased Haugh unit and eggshell color in brown eggs has a negative impact on egg sales and price. The eggshell color of brown eggs is an important eggshell quality parameter that influences consumer preference [1]. However, the eggshell color of brown eggs becomes lighter as the flock ages [2]. Eggshell color primarily depends on the pigment within the eggshell, and protoporphyrin IX has been identified as the main pigment in the shell of brown eggs, but trace amounts of biliverdin and its zinc chelates are also present [3]. Protoporphyrin IX is synthesized in the eggshell glands and then deposited onto the cuticle at the end of eggshell formation. It is an immediate precursor of heme, and heme synthesis is regulated by several genes, including aminolevulinic acid synthase-1 (ALAS1), coproporphyrinogen oxidase (CPOX), ferrochelatase (FECH), ATP-binding cassette subfamily G member 2 (ABCG2) and feline leukemia virus subgroup C receptor (FLVCR) [4].

The Haugh unit, which is calculated based on the albumen height and egg weight, is an important indicator of albumen quality [5], and it decreases as the age of laying hens increases [6]. A previous study compared the Haugh unit of eggs from laying hens at 44, 64 and 73 wk of age, and found that the Haugh unit exhibited a significant decline with age of laying hens [7]. Similarly, other reports have also found that the albumen height and Haugh unit of eggs produced by old laying hens were significantly lower than by young hens [8]. Albumen height mainly depends on the content of ovomucin synthesized in the magnum, which consists of α-ovomucin (OVOA) and β-ovomucin (OVOB) [9]. It interacts with other albumen proteins such as lysozyme (LYZ) and ovalbumin (OVAL) to form the ovomucin complex, an important component in maintaining the viscoelasticity of albumen [10]. Previous studies have shown a positive correlation between ovomucin content and Haugh unit in eggs during storage [11].

Although it has been shown that eggshell color and Haugh unit are associated with the pigments in eggshells and the ovomucin in eggs, the age-dependent changes of eggshell pigments and ovomucin in eggs have not been fully investigated. In addition, previous studies have shown that as the age of laying hens increases, their antioxidant function gradually declines [12], which may be an important reason for the deterioration of eggshell color and Haugh unit.

In light of the aforementioned findings, this study was thus conducted to compare egg quality, eggshell pigment and egg ovomucin content, antioxidant capacity, and the mRNA expression of genes related to pigment synthesis and albumen protein synthesis in laying hens with different ages.

## MATERIALS AND METHODS

### Animals, diet, experiment design, and housing

A total of 192 Hyline Brown laying hens at 165 (D165 group), 307 (D307 group) or 475 (D475 group) day of age provided by a local farm were allocated into three groups with eight replicates of eight hens each. All birds were from the same parental generation. They were all transferred to the same house for cage rearing, and were fed with the same diet for three weeks. The ingredient composition and nutrient level of basal diet are presented in Table 1. The hens were exposed to a 16:8 light: dark cycle, and they were allowed free access to mash feed and water.

### Sample collection

At the end of the experiment, three fresh eggs were randomly selected from each replicate for egg quality determination. Then one bird per replicate was randomly selected and killed by cervical dislocation. Blood (10 mL each) was taken from jugular vein and centrifuged at 4,000×g for 15 min at 4°C. Then the serum was separated and frozen at −20°C for further analysis. The eggshell gland and magnum samples were taken and frozen in liquid nitrogen until analysis.

### Egg quality assay

Eggshell strength was measured by the compression tester (Model-II; Robotmation). Egg albumen height, Haugh unit, and yolk color were measured by the egg multi-tester (EMT-5200; Robotmation). Eggshell color was measured with a colorimeter (CR-10; Konica Minolta) using the CIELAB system (L*, a*, b*) at three points (blunt end, equator, and sharp end). The yolk, albumen, and shell were separated and weighed to calculate their percentages of egg weight. Eggshell thickness was measured by a micrometer at the above three points after removing the eggshell membrane. Then the albumen and eggshell were stored at −20°C until analysis.

### Assay of eggshell pigment content

The protoporphyrin IX and biliverdin content of eggshell was measured as previously reported [13]. The eggshells collected above were cleaned and the eggshell membranes were removed. Subsequently, the cleaned eggshells were dried and pulverized to obtain eggshell powder. About 0.25 g of eggshell powder was placed in a 10 mL centrifuge tube with 4 mL of solvent (methanol:concentrated HCl = 2:1) in the dark for 12 h, and then the tube was centrifuged at 1,300×g for 45 min. The absorbance of the supernatant was measured using a spectrophotometer (UV2550; Shimadzu) at wavelengths of 412 and 670 nm, which represents the content of protoporphyrin and biliverdin, respectively.

### Assay of ovomucin content

The content of ovomucin was determined according to the methods as previously reported [14]. Triple volume of 0.1 mol/L NaCl solution was added to the blended albumen mixture and stirred at 350 r/min for 40 min at 4°C using a magnetic stirrer. And then the pH of the mixed solution was adjusted to 6.0 with 2 mol/L HCl and stirred for 30 min at 4°C. The dispersion was kept overnight at 4°C and separated by centrifugation at 15,000×g for 15 min at 4°C. The precipitate was suspended in 0.5 mol/L NaCl solution and then kept at 4°C for 2 h. After centrifugation at 15,000×g for 20 min at 4°C, the precipitate was rinsed three times with distilled water, and then was centrifuged for 15 min at 4°C and 15,000×g. The washed precipitates were dried in a vacuum freeze dryer (LGJ-25C; Beijing Fourring Scientific Instrument) for 48 h and weighed, and the ovomucin content was calculated.

### Determination of antioxidant capacity

The assay of total antioxidant capacity (T-AOC), total superoxide dismutase (T-SOD), glutathione (GSH) and malondialdehyde (MDA) in serum, eggshell gland and magnum was performed using analytical kits from Nanjing Jiancheng Bioengineering Institute. The results of above parameters in eggshell gland and magnum were normalized against corresponding total protein level measured by a bicinchoninic acid protein assay kit.

### Determination of mRNA expression

In order to preliminarily explore the underlying mechanism of the quality deterioration of eggs, real-time polymerase chain reaction (PCR) was used to determine the mRNA expression of genes related to pigment synthesis and transport (CPOX, FECH, ALAS1, ABCG2, and FLVCR) in eggshell gland, and genes related to albumen synthesis (OVAL, OVOA, OVOB, and LYZ) in magnum. Briefly, the total RNA was extracted from samples of eggshell gland and magnum using a SteadyPure RNA Extraction Kit (cat. AG21024; Accurate Biotechnology). The concentration and purity of total RNA were evaluated and quantified by a NanoDrop 2,000 spectrophotometer (Thermo Fisher Scientific), and total RNA was then reverse transcribed into cDNA using the Evo M-MLV RT Mix Kit (cat. AG11728). The cDNA was quantified using a SYBR Green Premix Pro Taq HS qPCR Kit (cat. AG11701) on the CFX Opus 96 RT-PCR System (Bio-Rad Laboratories). The RT-PCR programs were as follows: one cycle at 95°C for 30 s, 40 cycles at 95°C for 5 s, followed by a step of 60°C for 30 s. The primers for target genes were designed according to the sequences located in GenBank, and β-actin was used to normalize the target genes (Table 2). Relative mRNA levels (arbitrary units) were calculated on the basis of PCR efficiency and threshold cycle (Ct) values [15]. The mRNA level of each target gene for the D165 group was assigned a value of one.

### Statistical analysis

All data were analyzed by one-way ANOVA using SPSS 26.0 statistical software (IBM). The differences among groups were examined by Duncan’s multiple range test, which were considered to be significant at p<0.05. Data were presented as means and standard error of means.

## RESULTS

### Egg quality

Compared with the D165 group (Table 3), the eggshell L* value, eggshell ratio and egg yolk ratio in the D307 group were significantly increased (p<0.05), whereas the albumen ratio was significantly reduced (p<0.05). The eggshell L* value, eggshell b* value and egg yolk ratio in the D475 group were significantly increased (p<0.05), whereas albumen height, Haugh unit, eggshell a* value and albumen ratio were significantly decreased (p<0.05). However, there was no difference in eggshell strength and eggshell thickness among 3 groups.

### Eggshell pigment content

**Figure 1:**
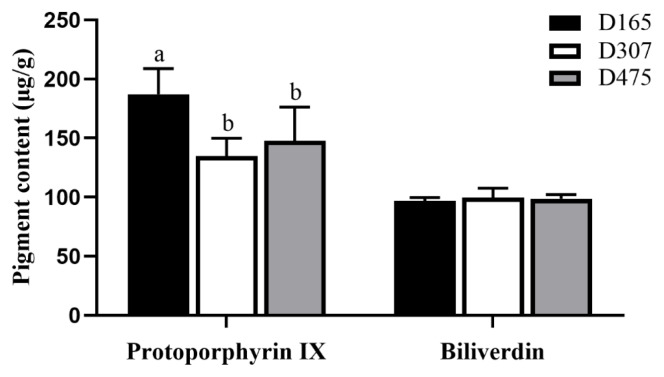
Content of protoporphyrin IX and biliverdin in eggshell of Hyline Brown laying hens. ^a,b^ Different letters above the error bar indicate significant difference at p<0.05. D165, 165-d-old laying hens; D307, 307-d-old laying hens; D475, 475-d-old laying hens.

Protoporphyrin IX content in eggshell of the D307 and D475 groups was significantly lower (p<0.05) than that in the D165 group (Figure 1), but there was no significant difference in the content of biliverdin in eggshells among 3 groups.

### Ovomucin content

**Figure 2:**
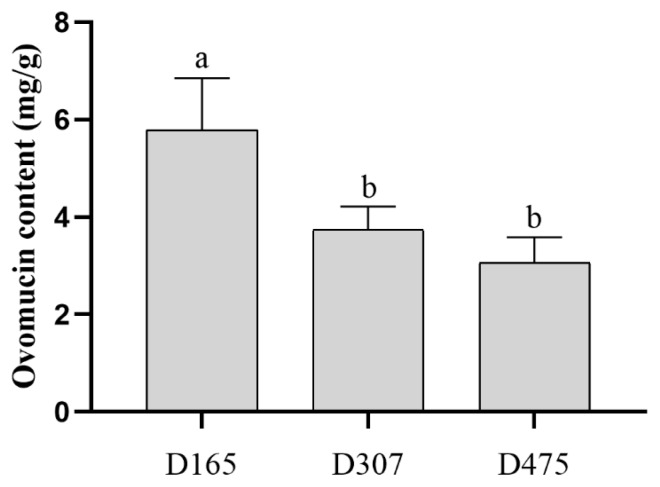
Egg ovomucin content of laying hens. ^a,b^ Different letters above the error bar indicate significant difference at p<0.05. D165, 165-day-old laying hens; D307, 307-day-old laying hens; D475, 475-day-old laying hens.

Ovomucin content of eggs in the D307 and D475 groups was lower (p<0.05) than in the D165 group (Figure 2), but no differences were observed between D307 and D525 groups. In addition, the ovomucin content of eggs showed a decreasing trend with the increase of age.

### Antioxidant capacity

The D307 and D475 groups showed higher (p<0.05) serum MDA level than the D165 group, but there was no difference in T-AOC, T-SOD and GSH (Table 4). In the eggshell gland, compared with the D165 group, the D307 group had lower (p<0.05) T-SOD activity and GSH level, and the D475 group had higher (p<0.05) MDA level. The T-SOD activity in the D307 group was also significantly lower than that in the D475 group. However, no significant effect was observed on T-AOC. In the magnum, the MDA levels in the D165 and D307 groups were lower than in the D475 group (p<0.05), but no difference was found in T-AOC, T-SOD and GSH.

### Messenger RNA expression

**Figure 3:**
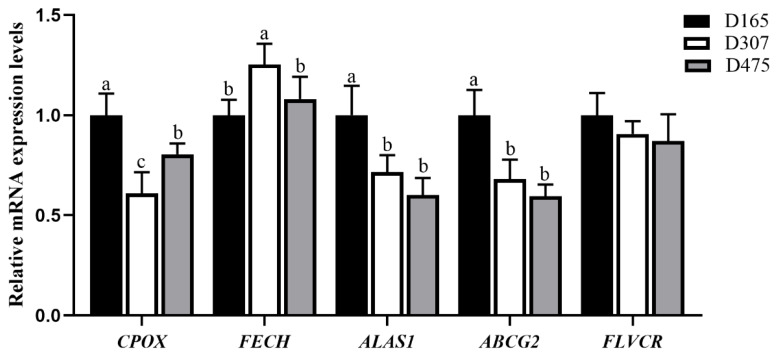
The mRNA levels of genes related to pigment synthesis and transport in eggshell gland of Hyline Brown laying hens. ^a–c^ Different letters above the error bar indicate significant difference at p<0.05. D165, 165-day-old laying hens; D307, 307-day-old laying hens; D475, 475-day-old laying hens; CPOX, coproporphyrinogen oxidase; FECH, ferrochelatase; ALAS1, aminolevulinic acid synthase-1; ABCG2, ATP-binding cassette subfamily G member 2; FLVCR, choline and heme transporter.

Compared with the D165 group, the D307 and D475 groups had lower (p<0.05) mRNA levels of CPOX, ALAS1 and ABCG2 in eggshell gland (Figure 3). The D307 group had lower CPOX mRNA level and higher FECH mRNA level than the other two groups (p<0.05). However, no significant difference was observed in the mRNA expression of FLVCR.

**Figure 4:**
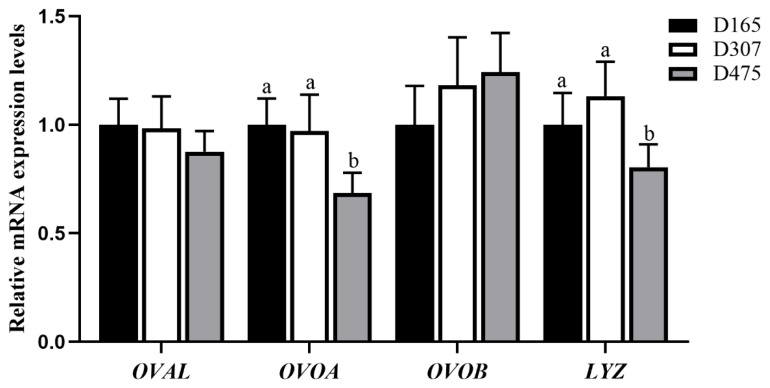
The mRNA levels of genes related to albumen synthesis in magnum of Hyline Brown laying hens. ^a,b^ Different letters above the error bar indicate significant difference at p<0.05. D165, 165-day-old laying hens; D307, 307-day-old laying hens; D475, 475-day-old laying hens; OVAL, ovalbumin; OVOA, α-ovomucin; OVOB, β-ovomucin; LYZ, lysozyme.

The D475 group had significantly lower (p<0.05) mRNA expression levels of OVOA and LYZ than the other two groups in the magnum (Figure 4). The mRNA levels of OVAL and OVOB in magnum were not affected by the age of laying hens.

## DISCUSSION

The judgment criteria for the lightening of the shell color of brown eggs are the increase of the L* value and the decrease of the a* value and b* value of the eggshell [16]. In the present study, the L* value of eggshells in the D307 and D475 groups was significantly higher than that in the D165 group, and the a* value of eggshells decreased significantly with the increase of age, agreeing with the results of previous studies [2]. All these results indicate that eggshell color gradually gets lighter as hen ages.

The eggshell color of brown eggs mainly depends on the content of protoporphyrin IX, an immediate precursor of heme [17]. It has been reported that the content of protoporphyrin IX in dark-colored eggshells is higher than that in light-colored eggshells [18]. In this experiment, the content of protoporphyrin IX in eggshells of D307 group and D475 group was significantly lower than that of D165 group, which was consistent with the changes of eggshell color. Our finding suggests that the age-dependent change of eggshell color may be due to the decreased deposition of protoporphyrin IX. A previous study also showed that the eggshell L* value was negatively correlated with protoporphyrin IX, and a* value was positively correlated with protoporphyrin IX [19]. However, there was no significant change in the biliverdin content in the eggshells of eggs, demonstrating that biliverdin content had almost no effect on the eggshell color of brown eggs [20].

The synthesis, transport, and deposition of protoporphyrin IX are controlled by multiple genes including ALAS1, CPOX, FECH and ABCG2 [21]. The ALAS1 is the first and rate-limiting enzyme in the heme synthesis pathway [22]. It has been reported that when the expression level of ALAS1 in the eggshell gland is downregulated, the content of protoporphyrin IX in both the eggshell gland and eggshell decreases [22], and the hens laying dark brown eggs had higher ALAS1 mRNA level in the eggshell gland than those laying light brown eggs [23], indicating that the expression of ALAS1 may affect the synthesis of protoporphyrin IX in the eggshell gland and eggshell color. CPOX is the rate limiting enzyme in heme synthesis during erythroid differentiation [24], which catalyzes the oxidative decarboxylation of coproporphyrinogen III to form protoporphyrinogen [25]. High levels of CPOX lead to increased synthesis of protoporphyrinogen, which in turn promotes the synthesis of protoporphyrin IX and darkens the eggshell color [22]. The FECH is also the rate limiting enzyme in heme synthesis, which catalyzes the binding of Fe^2+^ to protoporphyrin IX to form heme [26]. High levels of FECH convert more protoporphyrinogen into heme, reduce protoporphyrinogen deposition and generate a light eggshell color [25]. ABCG2 functions as an integral protein transporter during this biosynthetic process, mediating the export of intracellular protoporphyrin IX out of cells [27], and promoting the deposition of protoporphyrin IX in the eggshell gland. In this study, the D307 and the D475 group had lower mRNA levels of ALAS1, CPOX, and ABCG2 in the eggshell glands than the D165 group, which was consistent with previous research [18], indicating that the expression of genes related to protoporphyrin IX synthesis and transport decreased as hens aged. In addition, the D307 group had higher mRNA level of FECH in the eggshell glands than the other two groups, implying that more protoporphyrin IX is converted into heme in the D307 group. Therefore, this study speculates that hen age affects the gene expression levels of ALAS1, CPOX, FECH, and ABCG2, and then affects the synthesis, transport, and deposition of protoporphyrin IX in the eggshell gland, resulting in the change of eggshell color.

Haugh unit was observed to decrease with age in the present experiment, which was in agreement with previous studies [28]. This can be explained by the changes of ovomucin in eggs. The ovomucin content of the D307 and D475 groups was lower than that in the D165 group in this trial, suggesting that the amount of ovomucin in eggs decreases as the hen ages. Ovomucin is a key component in maintaining the viscosity of egg whites and determines their albumen height and Haugh unit [29]. There is a strong positive correlation between ovomucin content and Haugh unit [11].

In the daily cycle, egg white proteins, including OVO, OVAL, LYZ, are synthesized and secreted by the epithelial cells of the oviduct magnum [30]. Ovomucin contains two subunits, α and β, and egg white thinning has been found to be related to the breakdown of these two subunits in previous studies [31]. LYZ often exists in egg white in the form of a complex with ovomucin, and participates in maintaining the gel properties of egg whites [32]. Our results indicated that the mRNA expression of OVOA and LYZ genes was obviously down-regulated with age, indicating that aging affects Haugh unit by suppressing protein synthesis and secretion in magnum. It has been reported that aging can exacerbate the oxidative damage of the body, which leads to the imbalance of the redox system and causes the reduction of protein metabolic capacity [33]. Moreover, the utilization rate of nutrients and the ability to transport and absorb amino acids in laying hens also decrease with age [34]. Both of them may be the reason for the change in the expression level of above genes. There was no significant difference in OVAL mRNA level in the magnum of laying hens in this experiment. However, a previous study found that the expression of OVAL gene in the oviduct of laying hens decreased during aging [35]. The discrepancy might be related to the breed and age of laying hens. In their study, mature egg-laying and old non-egg-laying hens were used, which was different from our study.

Many studies have shown that oxidative damage is a major factor in the decline in physiologic function that occurs during the aging process. A study found that old hens (580 d) had lower T-AOC, T-SOD activity and GSH content in ovary than young hens (90, 150 and 280 d), indicating that the antioxidant capacity of the ovaries decreases during aging [36]. In this study, the T-AOC in serum showed a downward trend with age, and the D307 group had lower T-SOD activity and GSH content in the eggshell gland than the D165 group, suggesting that the antioxidant capacity of hens declined with age. Moreover, the D475 group had higher MDA content in the serum, eggshell gland, and magnum than the D165 group. This indicates that aging promotes lipid peroxidation and has a negative impact on the redox system [37]. However, the current results show that the T-SOD activity in the eggshell gland of the D475 group was higher than that of the D307 group, which was hard to explain. The above results indicated that the antioxidant capacity of laying hens decreased and increased oxidative stress occurred during the aging process. Previous studies have found that the decline in ovarian function in mice is related to oxidative damage caused by aging [38]. And in other studies, it has also been found that with the increase of age, the antioxidant capacity of laying hens degenerates and the redox balance is disrupted [39]. This imbalance severely impairs reproductive performance, leads to tissue damage, and causes the decline in egg production performance in laying hens [40]. Therefore, it can be inferred that the decrease in the antioxidant capacity of the eggshell gland and the magnum caused by aging also has an adverse effect on the synthesis and secretion functions of the eggshell gland and the magnum, affecting the deposition of protoporphyrin IX and the secretion of ovomucin.

## CONCLUSION

In conclusion, aging resulted in lighter eggshell color and lower Haugh unit of eggs in laying hens, owing to decreased content of protoporphyrin IX and ovomucin and related gene expression in the eggshell gland and magnum, which was associated with increased degree of lipid peroxidation.

## Figures and Tables

**Table 1:** Ingredient composition and nutrient content of the basal diet (as-fed basis, g·kg^−1^ unless otherwise stated)

Item	Content
Ingredient	
Corn	645
Soybean meal	230
Soybean oil	5
Limestone	95
Premix^1)^	25
Total	1,000
Calculated nutrient composition	
Metabolizable energy (MJ/kg)	11.16
Crude protein	153.3
Lysine	7.7
Methionine	3.4
Methionine+cystine	6.0
Calcium	38.9
Available phosphorus	2.7

1) Premix supplied per kilogram of diet: transretinyl acetate, 11,000 IU; cholecalciferol, 3,500 IU; all-rac-α-tocopherol acetate, 20 mg; menadione, 1.5 mg; thiamin, 1 mg; riboflavin, 6 mg; nicotinamide, 40 mg; choline chloride, 400 mg; calcium pantothenate, 10 mg; pyridoxine·HCl, 2 mg; biotin, 0.04 mg; folic acid, 1 mg; cobalamin, 0.02 mg; Fe, 60 mg; Cu, 8 mg; Mn, 100 mg; Zn, 65 mg; I, 1 mg; Se, 0.3 mg; Ca, 4 g; P, 2 g; Met, 1 g; sodium chloride, 2 g.

**Table 2:** Sequences for real-time PCR primers

Gene	GenBank number	Primer sequence, sense/antisense
β-Actin	NM_205518.2	TATGTGCAAGGCCGGTTTC
		TGTCTTTCTGGCCCATACCAA
CPOX	XM_040660202.2	GCTCCGTTATCCATCCAA
		CACCACCAAACCACCACT
FECH	XM_046934645.1	CCAGTGTTTGCCGATCACATAC
		TCACCACGGTTCACCACAGACAT
ALAS1	NM_001018012.2	GGTGGACAGGAAAGGTAAAGA
		ACTGGTCATACTGGAAGGTG
ABCG2	XM_046920216.1	TATCAGTGCTGGGATGGAAGTG
		GAAGGGAGGTTTACAAGGAGGC
FLVCR	XM_040667192.2	AAGAAGATGGCTTAGAAACGC
		ACTGTCACCAAAGTCACGAAA
OVAL	NM_205152.3	GCACTCAAGCTCAAAAGACAACT
		ATCTGTGTCCTGGTGCTGTC
OVOA	XM_046917694.1	GGACCAGAAGACGACCGTTGTTG
		GCATAAGCAGCCACCGAGGTAC
OVOB	XM_046917979.1	CCCTAACAACCAGTGCTCCAACAG
		TGGCAGTAGTCCTCTCCGTGATG
LYZ	NM_205281.2	FTACGACACTGGCAACATGAG
		CGGCTGTTGATCTGTAGGATT

PCR, polymerase chain reaction; CPOX, coproporphyrinogen oxidase; FECH, ferrochelatase; ALAS1, aminolevulinic acid synthase-1; ABCG2, ATP-binding cassette subfamily G member 2; FLVCR, choline and heme transporter; OVAL, ovalbumin; OVOA, α-ovomucin; OVOB, β-ovomucin; LYZ, lysozyme.

**Table 3:** Effect of age on egg quality of Hyline Brown laying hens

Items	Treatments			SEM	p-value

	D165	D307	D475		
Albumen height (mm)	11.36^a^	10.45^ab^	9.46^b^	0.31	0.029
Haugh unit	104.43^a^	100.59^ab^	94.87^b^	1.48	0.019
Eggshell strength (N)	40.22	51.85	40.27	2.34	0.067
Eggshell thickness (μm)	371.81	400.46	356.93	7.84	0.071
Eggshell L* value	39.39^b^	44.48^a^	44.67^a^	0.73	0.001
Eggshell a* value	26.92^a^	24.38^b^	20.93^c^	0.65	<0.001
Eggshell b* value	40.80^b^	40.62^b^	49.12^a^	1.13	<0.001
Eggshell ratio (%)	12.45^b^	13.86^a^	12.05^b^	0.27	0.011
Egg yolk ratio (%)	21.76^b^	26.72^a^	26.23^a^	0.57	<0.001
Albumen ratio (%)	65.78^a^	59.41^b^	61.72^b^	0.69	<0.001

a–c Values within a row with different superscripts differ significantly at p<0.05.

D165, 165-day-old laying hens; D307, 307-day-old laying hens; D475, 475-day-old laying hens; SEM, standard error of the mean.

**Table 4:** Effect of age on antioxidant indicators of Hyline Brown laying hens

Items	Treatments			SEM	p-value

	D165	D307	D475		
Serum					
T-AOC (μmol/L)	426.85	340.38	395.65	15.37	0.060
T-SOD (U/mL)	524.59	541.93	555.53	12.42	0.615
GSH (mg/mL)	6.00	7.28	5.90	0.30	0.110
MDA (nmol/mL)	1.88^b^	2.48^a^	2.34^a^	0.08	0.001
Eggshell gland					
T-AOC (μmol/g protein)	147.37	114.82	126.42	7.11	0.159
T-SOD (U/mg protein)	328.51^a^	280.01^b^	322.87^a^	8.45	0.030
GSH (mg/g protein)	5.27^a^	3.86^b^	4.59^ab^	0.21	0.021
MDA (nmol/mg protein)	1.45^b^	1.58^ab^	1.91^a^	0.08	0.036
Magnum					
T-AOC (μmol/g protein)	30.95	27.15	29.08	1.15	0.422
T-SOD (U/mg protein)	62.24	69.80	71.50	2.48	0.281
GSH (mg/g protein)	1.59	1.27	1.62	0.07	0.074
MDA (nmol/mg protein)	0.28^b^	0.33^b^	0.46^a^	0.03	0.014

a,b Values within a row with different superscripts differ significantly at p<0.05.

D165, 165-day-old laying hens; D307, 307-day-old laying hens; D475, 475-day-old laying hens; SEM, standard error of the mean; T-AOC, total antioxidant capacity; T-SOD, total superoxide dismutase; GSH, glutathione; MDA, malondialdehyde.

## Data Availability

Upon reasonable request, the datasets of this study can be available from the corresponding author.
